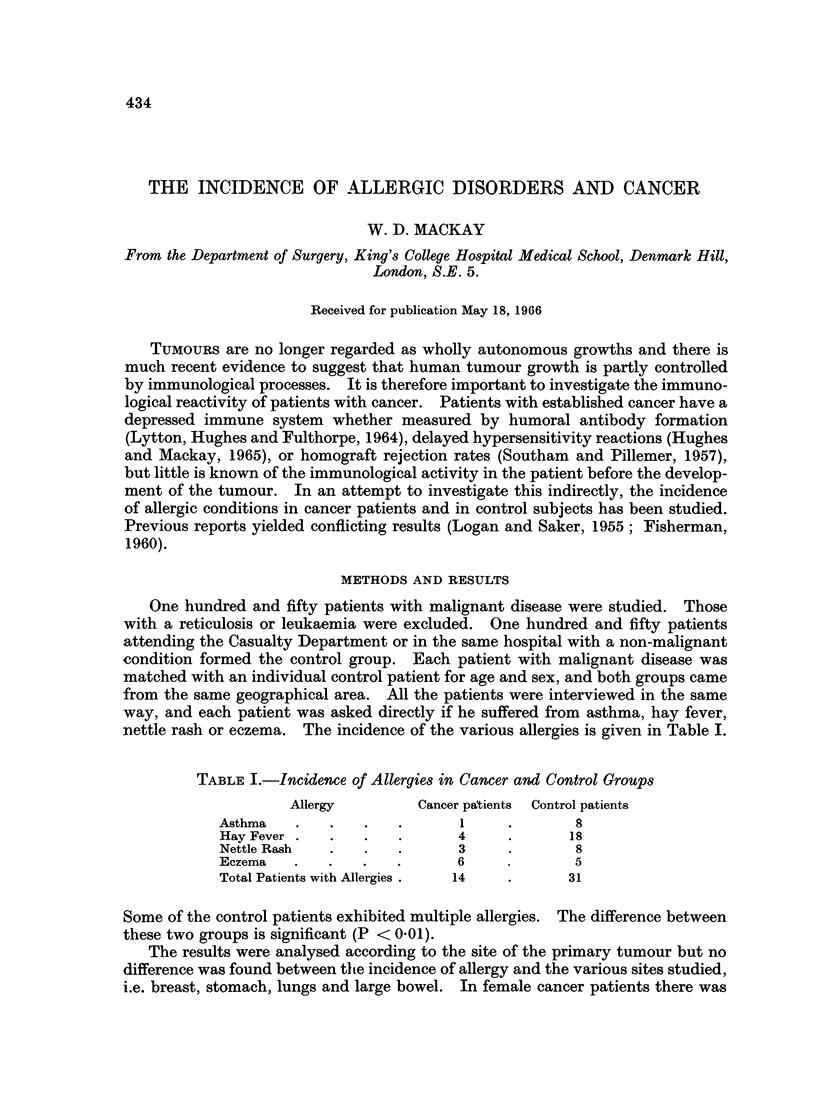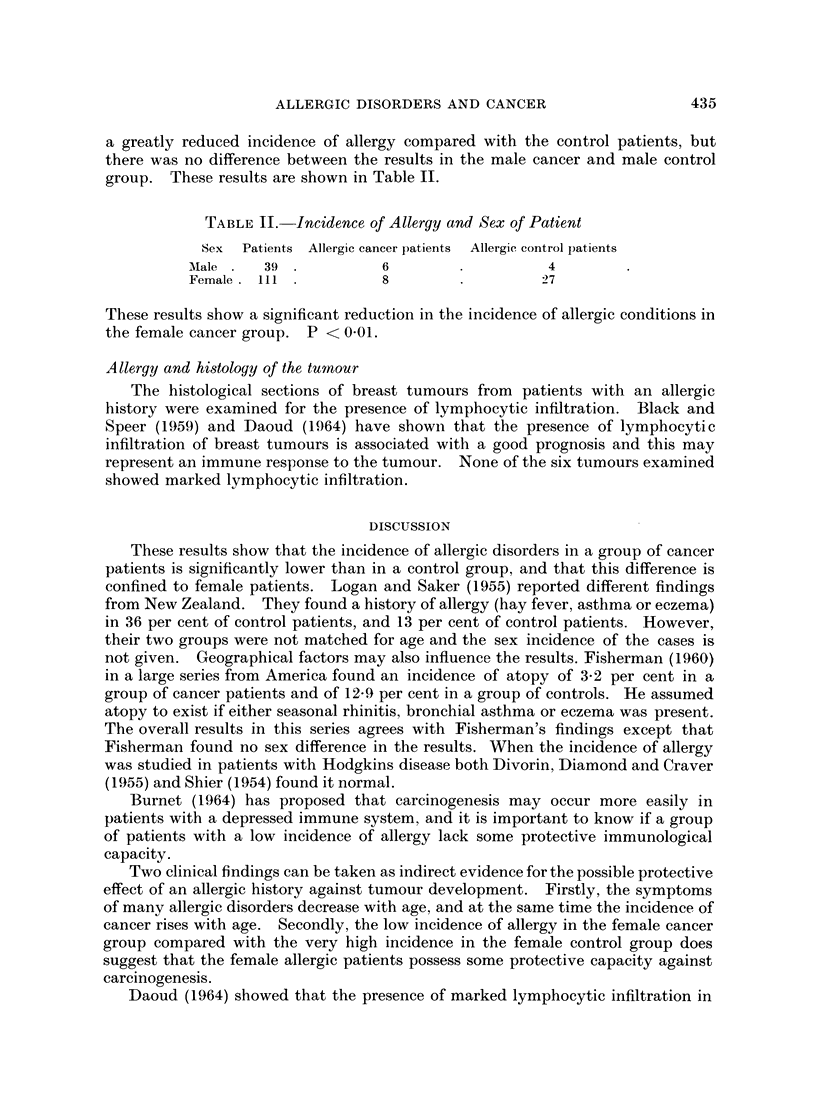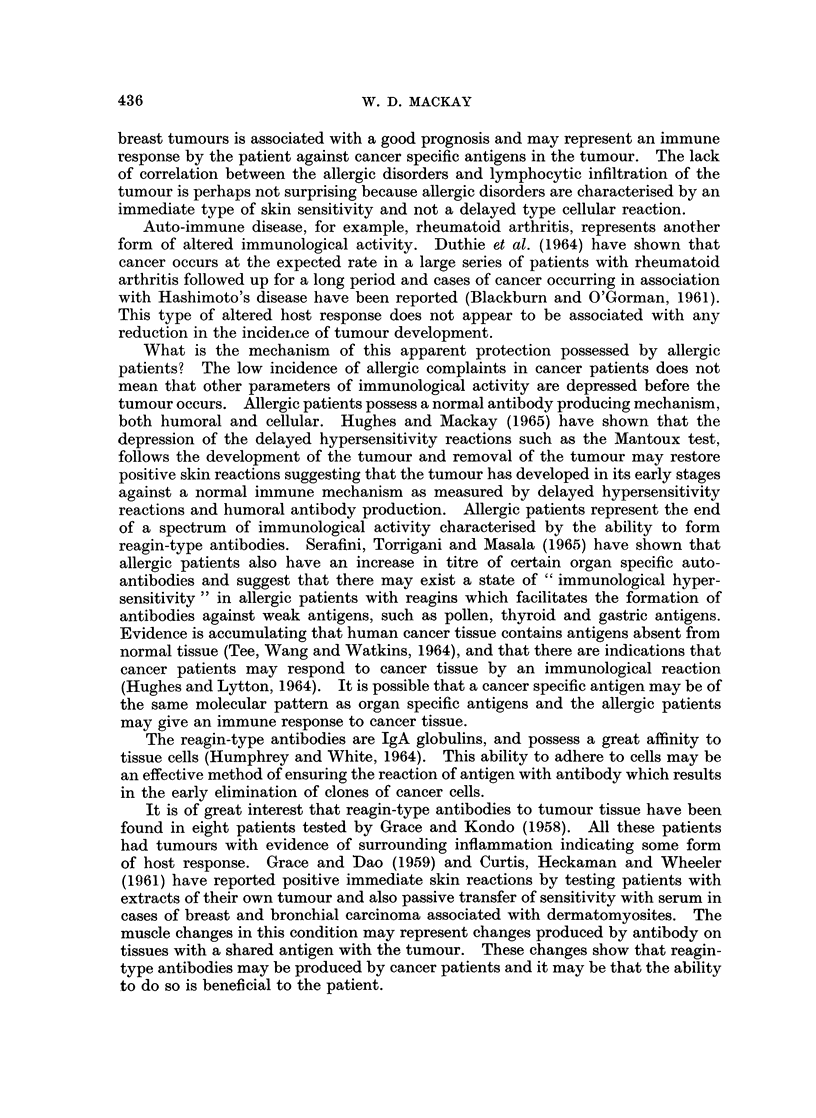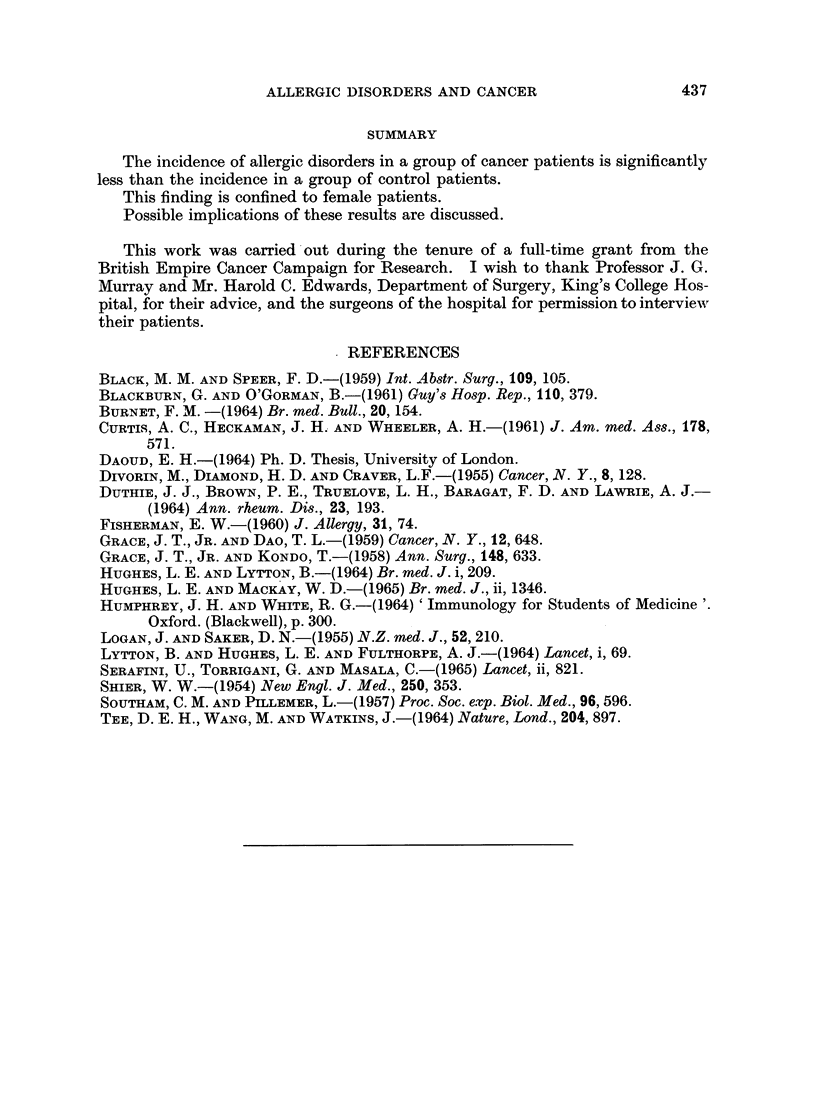# The incidence of allergic disorders and cancer.

**DOI:** 10.1038/bjc.1966.52

**Published:** 1966-09

**Authors:** W. D. Mackay


### THE INCIDENCE OF ALLERGIC DISORDERS AND CANCER

W. D. MACKAY

*From the Department of Surgery, King's College Hospital Medical School, Denmark Hill, London, S.E. 5.*

Received for publication May 18, 1966

TUMOURS are no longer regarded as wholly autonomous growths and there is much recent evidence to suggest that human tumour growth is partly controlled by immunological processes. It is therefore important to investigate the immunological reactivity of patients with cancer. Patients with established cancer have a depressed immune system whether measured by humoral antibody formation (Lytton, Hughes and Fulthorpe, 1964), delayed hypersensitivity reactions (Hughes and Mackay, 1965), or homograft rejection rates (Southam and Pillemer, 1957), but little is known of the immunological activity in the patient before the development of the tumour. In an attempt to investigate this indirectly, the incidence of allergic conditions in cancer patients and in control subjects has been studied. Previous reports yielded conflicting results (Logan and Saker, 1955 ; Fisherman, 1960).

### METHODS AND RESULTS

One hundred and fifty patients with malignant disease were studied. Those with a reticulosis or leukaemia were excluded. One hundred and fifty patients attending the Casualty Department or in the same hospital with a non-malignant condition formed the control group. Each patient with malignant disease was matched with an individual control patient for age and sex, and both groups came from the same geographical area. All the patients were interviewed in the same way, and each patient was asked directly if he suffered from asthma, hay fever, nettle rash or eczema. The incidence of the various allergies is given in Table I.

TABLE I.—*Incidence of Allergies in Cancer and Control Groups*

| Allergy | Cancer patients | Control patients |
|---|---|---|
| Asthma | 1 | 8 |
| Hay Fever | 4 | 18 |
| Nettle Rash | 3 | 8 |
| Eczema | 6 | 5 |
| Total Patients with Allergies | 14 | 31 |

Some of the control patients exhibited multiple allergies. The difference between these two groups is significant ($P < 0{\cdot}01$).

The results were analysed according to the site of the primary tumour but no difference was found between the incidence of allergy and the various sites studied, i.e. breast, stomach, lungs and large bowel. In female cancer patients there was a greatly reduced incidence of allergy compared with the control patients, but there was no difference between the results in the male cancer and male control group. These results are shown in Table II.

TABLE II.—*Incidence of Allergy and Sex of Patient*

| Sex | Patients | Allergic cancer patients | Allergic control patients |
|---|---|---|---|
| Male | 39 | 6 | 4 |
| Female | 111 | 8 | 27 |

These results show a significant reduction in the incidence of allergic conditions in the female cancer group. $P < 0.01$.

*Allergy and histology of the tumour*

The histological sections of breast tumours from patients with an allergic history were examined for the presence of lymphocytic infiltration. Black and Speer (1959) and Daoud (1964) have shown that the presence of lymphocytic infiltration of breast tumours is associated with a good prognosis and this may represent an immune response to the tumour. None of the six tumours examined showed marked lymphocytic infiltration.

### DISCUSSION

These results show that the incidence of allergic disorders in a group of cancer patients is significantly lower than in a control group, and that this difference is confined to female patients. Logan and Saker (1955) reported different findings from New Zealand. They found a history of allergy (hay fever, asthma or eczema) in 36 per cent of control patients, and 13 per cent of control patients. However, their two groups were not matched for age and the sex incidence of the cases is not given. Geographical factors may also influence the results. Fisherman (1960) in a large series from America found an incidence of atopy of 3·2 per cent in a group of cancer patients and of 12·9 per cent in a group of controls. He assumed atopy to exist if either seasonal rhinitis, bronchial asthma or eczema was present. The overall results in this series agrees with Fisherman's findings except that Fisherman found no sex difference in the results. When the incidence of allergy was studied in patients with Hodgkins disease both Divorin, Diamond and Craver (1955) and Shier (1954) found it normal.

Burnet (1964) has proposed that carcinogenesis may occur more easily in patients with a depressed immune system, and it is important to know if a group of patients with a low incidence of allergy lack some protective immunological capacity.

Two clinical findings can be taken as indirect evidence for the possible protective effect of an allergic history against tumour development. Firstly, the symptoms of many allergic disorders decrease with age, and at the same time the incidence of cancer rises with age. Secondly, the low incidence of allergy in the female cancer group compared with the very high incidence in the female control group does suggest that the female allergic patients possess some protective capacity against carcinogenesis.

Daoud (1964) showed that the presence of marked lymphocytic infiltration in breast tumours is associated with a good prognosis and may represent an immune response by the patient against cancer specific antigens in the tumour. The lack of correlation between the allergic disorders and lymphocytic infiltration of the tumour is perhaps not surprising because allergic disorders are characterised by an immediate type of skin sensitivity and not a delayed type cellular reaction.

Auto-immune disease, for example, rheumatoid arthritis, represents another form of altered immunological activity. Duthie *et al.* (1964) have shown that cancer occurs at the expected rate in a large series of patients with rheumatoid arthritis followed up for a long period and cases of cancer occurring in association with Hashimoto's disease have been reported (Blackburn and O'Gorman, 1961). This type of altered host response does not appear to be associated with any reduction in the incidence of tumour development.

What is the mechanism of this apparent protection possessed by allergic patients? The low incidence of allergic complaints in cancer patients does not mean that other parameters of immunological activity are depressed before the tumour occurs. Allergic patients possess a normal antibody producing mechanism, both humoral and cellular. Hughes and Mackay (1965) have shown that the depression of the delayed hypersensitivity reactions such as the Mantoux test, follows the development of the tumour and removal of the tumour may restore positive skin reactions suggesting that the tumour has developed in its early stages against a normal immune mechanism as measured by delayed hypersensitivity reactions and humoral antibody production. Allergic patients represent the end of a spectrum of immunological activity characterised by the ability to form reagin-type antibodies. Serafini, Torrigani and Masala (1965) have shown that allergic patients also have an increase in titre of certain organ specific auto-antibodies and suggest that there may exist a state of " immunological hypersensitivity " in allergic patients with reagins which facilitates the formation of antibodies against weak antigens, such as pollen, thyroid and gastric antigens. Evidence is accumulating that human cancer tissue contains antigens absent from normal tissue (Tee, Wang and Watkins, 1964), and that there are indications that cancer patients may respond to cancer tissue by an immunological reaction (Hughes and Lytton, 1964). It is possible that a cancer specific antigen may be of the same molecular pattern as organ specific antigens and the allergic patients may give an immune response to cancer tissue.

The reagin-type antibodies are IgA globulins, and possess a great affinity to tissue cells (Humphrey and White, 1964). This ability to adhere to cells may be an effective method of ensuring the reaction of antigen with antibody which results in the early elimination of clones of cancer cells.

It is of great interest that reagin-type antibodies to tumour tissue have been found in eight patients tested by Grace and Kondo (1958). All these patients had tumours with evidence of surrounding inflammation indicating some form of host response. Grace and Dao (1959) and Curtis, Heckaman and Wheeler (1961) have reported positive immediate skin reactions by testing patients with extracts of their own tumour and also passive transfer of sensitivity with serum in cases of breast and bronchial carcinoma associated with dermatomyosites. The muscle changes in this condition may represent changes produced by antibody on tissues with a shared antigen with the tumour. These changes show that reagin-type antibodies may be produced by cancer patients and it may be that the ability to do so is beneficial to the patient.

SUMMARY

The incidence of allergic disorders in a group of cancer patients is significantly less than the incidence in a group of control patients.

This finding is confined to female patients.

Possible implications of these results are discussed.

This work was carried out during the tenure of a full-time grant from the British Empire Cancer Campaign for Research. I wish to thank Professor J. G. Murray and Mr. Harold C. Edwards, Department of Surgery, King's College Hospital, for their advice, and the surgeons of the hospital for permission to interview their patients.

### REFERENCES

BLACK, M. M. AND SPEER, F. D.—(1959) *Int. Abstr. Surg.*, **109**, 105.
BLACKBURN, G. AND O'GORMAN, B.—(1961) *Guy's Hosp. Rep.*, **110**, 379.
BURNET, F. M. —(1964) *Br. med. Bull.*, **20**, 154.
CURTIS, A. C., HECKAMAN, J. H. AND WHEELER, A. H.—(1961) *J. Am. med. Ass.*, **178**, 571.
DAOUD, E. H.—(1964) Ph. D. Thesis, University of London.
DIVORIN, M., DIAMOND, H. D. AND CRAVER, L.F.—(1955) *Cancer, N. Y.*, **8**, 128.
DUTHIE, J. J., BROWN, P. E., TRUELOVE, L. H., BARAGAT, F. D. AND LAWRIE, A. J.—(1964) *Ann. rheum. Dis.*, **23**, 193.
FISHERMAN, E. W.—(1960) *J. Allergy*, **31**, 74.
GRACE, J. T., JR. AND DAO, T. L.—(1959) *Cancer, N. Y.*, **12**, 648.
GRACE, J. T., JR. AND KONDO, T.—(1958) *Ann. Surg.*, **148**, 633.
HUGHES, L. E. AND LYTTON, B.—(1964) *Br. med. J.* i, 209.
HUGHES, L. E. AND MACKAY, W. D.—(1965) *Br. med. J.*, ii, 1346.
HUMPHREY, J. H. AND WHITE, R. G.—(1964) ' Immunology for Students of Medicine '. Oxford. (Blackwell), p. 300.
LOGAN, J. AND SAKER, D. N.—(1955) *N.Z. med. J.*, **52**, 210.
LYTTON, B. AND HUGHES, L. E. AND FULTHORPE, A. J.—(1964) *Lancet*, i, 69.
SERAFINI, U., TORRIGANI, G. AND MASALA, C.—(1965) *Lancet*, ii, 821.
SHIER, W. W.—(1954) *New Engl. J. Med.*, **250**, 353.
SOUTHAM, C. M. AND PILLEMER, L.—(1957) *Proc. Soc. exp. Biol. Med.*, **96**, 596.
TEE, D. E. H., WANG, M. AND WATKINS, J.—(1964) *Nature, Lond.*, **204**, 897.